# Horseshoe kidney

**DOI:** 10.11604/pamj.2018.30.26.14899

**Published:** 2018-05-15

**Authors:** Guglielmo Mantica, Hilgard Ackermann

**Affiliations:** 1 Department of Urology, Stellenbosch University and Tygerberg Hospital, Cape Town, South Africa; 2 Department of Urology, Ospedale Policlinico San Martino, University of Genoa, Genoa, Italy

**Keywords:** Horseshoe kidney, ureteral obstruction, urinary tract infections

## Image in medicine

A 21-year-old male presented with a one day history of right flank pain and signs of severe sepsis. The recorded vital signs included a temperature of 39.9° Celsius, blood pressure of 95/65 mmHg and heart rate of 165. The laboratory investigations confirmed a raised C-reactive protein (CRP) of 485 mg/l but it was interesting to note thatthe white cell count and creatinine were respectively normal (5.85 x 10^9^/l; 77 µmol/l). Microbiological investigations confirmed Klebsiella pneumoniae (>10^6^ cfu/mL) on urine culture whilst two blood cultures were negative. Intravenous fluid resuscitation was successful in restoring hemodynamic stability. Amoxicillin/clavulanic acid (1200mg) was administered and continued for seven days after confirmation on antibiotic sensitivity testing. After ultrasonography confirmed severe right hydronephrosis, a contrasted computed tomography (CT) scan confirmed a horseshoe kidney (A, B, C, D) with an obstructed right-sided renalmoiety secondary to right pelvi-ureteric junction (PUJ) obstruction (B) and urothelial enhancement suggestive of an infected collecting system. Horseshoe kidneys are the most common congenital renal abnormality with an incidence of 1:400-600. It is commonly asyptomatic and rarely require interventionunless treating complications such as ureteral obstruction (PUJ), urinary tract infections, urolithiasis andWilms Tumour. It is associated with Trisomy 18 and females with Turner syndrome. Our patient underwent a right percutaneous nephrostomy (PCN) and 500 millilitres of infected urine were aspirated. He made a complete recovery at discharge. An interval MAG3 renogram confirmed that both moeities had good and excretion. A pelvi-ureteric junction repair will be performed after 6 weeks.

**Figure 1:**
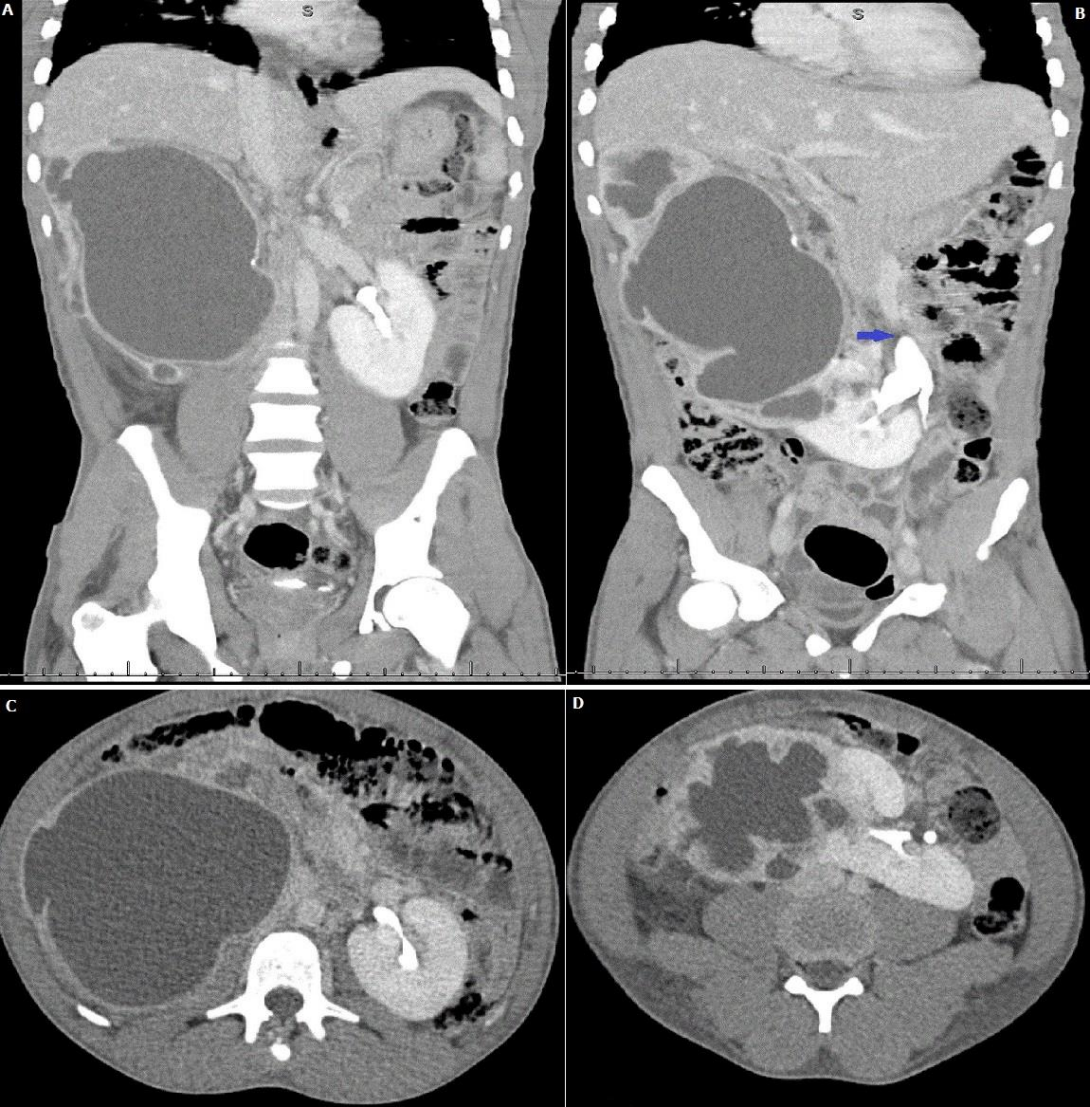
CT-IVP coronal (A, B), axial (C, D) images of the horseshoe kidney, the blue arrow labels the PUJ obstruction

